# Structural insight into β-Clamp and its interaction with DNA Ligase in *Helicobacter pylori*

**DOI:** 10.1038/srep31181

**Published:** 2016-08-08

**Authors:** Preeti Pandey, Khaja Faisal Tarique, Mohit Mazumder, Syed Arif Abdul Rehman, Nilima kumari, Samudrala Gourinath

**Affiliations:** 1 School of Life Sciences, Jawaharlal Nehru University, New Delhi, India; 2 Department of Bioscience and Biotechnology, Banasthali University, Rajasthan, India

## Abstract

*Helicobacter pylori,* a gram-negative and microaerophilic bacterium, is the major cause of chronic gastritis, gastric ulcers and gastric cancer. Owing to its central role, DNA replication machinery has emerged as a prime target for the development of antimicrobial drugs. Here, we report 2Å structure of β-clamp from *H. pylori* (Hpβ-clamp), which is one of the critical components of DNA polymerase III. Despite of similarity in the overall fold of eubacterial β-clamp structures, some distinct features in DNA interacting loops exists that have not been reported previously. The *in silico* prediction identified the potential binders of β-clamp such as alpha subunit of DNA pol III and DNA ligase with identification of β-clamp binding regions in them and validated by SPR studies. Hpβ-clamp interacts with DNA ligase in micromolar binding affinity. Moreover, we have successfully determined the co-crystal structure of β-clamp with peptide from DNA ligase (not reported earlier in prokaryotes) revealing the region from ligase that interacts with β-clamp.

The sliding clamp is a ring-shaped protein complex that encircles DNA with the help of clamp loader in an ATP-dependent manner, and slides along the DNA. Because of its ability to slide along DNA, the sliding clamp is required by many different enzymes for DNA replication and repair^1^. Clamps not only increase the processivity of these enzymes but also serve as attachment points to coordinate their activities. The clamps are thus required for keeping these enzymes tightly associated with DNA while at the same time facilitating their translocation along duplex DNA^2^.

The elongation factor β-clamp also called sliding clamp has been found to exist in both prokaryotes and eukaryotes. In eukaryotes, it is generally known by the name PCNA and is a heterotrimer. Each monomer consists of two domains, with N-terminal domain joint to C-terminal domain of neighboring monomers by non-covalent interactions and form a ring-shaped structure^3^. In prokaryotes, however, β-clamp is a homodimer, with each monomer consisting of three globular domains, and in this way β-clamp displays a six-domain ring^4^. Thus despite of having sequence similarity between these two, they share similar architecture as suggested by their structural analysis^3^,^5^. All of the known clamp-binding proteins contain a conserved peptide sequence motif through which they interact with the clamp^6^. In both prokaryotes and eukaryotes, a key feature of this clamp-binding motif is the presence of hydrophobic amino acid residues that bind to the hydrophobic pocket in the C-terminal region of the clamp. Based on experimental studies, QL(S/D)LF^7^ and QxxL(x)F^8^ have been proposed as consensus binding sequences for *E. coli*β-clamp.

Although β-clamp is part of the DNA polymerase III holoenzyme, it is not attached to polymerase III permanently like the other subunits. β-clamp is loaded on the DNA, by clamp loader, a subunit of DNA Pol III. It interacts with several proteins other than DNA polymerase III subunits; it also freely slides along DNA and improves the processivity of other proteins. Among the several β-clamp-interacting partners, one of the most important protein is DNA ligase. After completion of the synthesis of the lagging strand fragment, DNA polymerase III becomes separated from β-clamp and DNA and moves to another primed site^9^. It was hypothesized that this released beta clamp interacts with Pol I, which digests RNA primer at the 5′ end of the primer and replaces it with DNA by nick translation. After that, the clamp interacts with DNA ligase, which seals the nick^10^. Thus, the interaction between β-clamp and DNA ligase helps in Okazaki fragment maturation, and is also needed for DNA repair. Therefore, studying the interaction between these two components is of great importance. In case of prokaryotes nothing much is known about β-clamp – DNA ligase interaction except for the existence of binding in case of *E. coli*^10^ and absent in *M. tuberculosis*^11^. Despite of having similar catalytic mechanism, the structure of prokaryotic DNA ligase is little bit different from that of eukaryotic in having four domains – an adenylation domain, oligomer-binding (OB) fold, Zinc finger & helix-hairpin-helix and the last one BRCT (breast cancer carboxy-terminal) domain.

*Helicobacter pylori* is a gram-negative, microaerophilic bacterium, mainly found in the stomach, and is a major cause of chronic gastritis and gastric ulcers, and leads to stomach cancer and gastric carcinoma, which constitute the second most common type of cancer in the world^12^. One of the most striking characteristics of *H. pylori* biology is its remarkable allelic diversity and genetic variability. It undergoes genetic alterations *in vivo*, resulting from an elevated mutation rate and frequent intraspecific recombination^13^. This property of *H. pylori* makes it different from other bacteria. Also, the functions many proteins in general—and of replication initiation proteins in particular —are different in *H. pylori* than in other bacteria. For example, in *H. pylori*, helicase loader is not present^14^, and helicase and primase interact with each other very strongly^15^,^16^; these properties are quite different from those of *E. coli*. The β-clamp and ligase both are important proteins in replication machinery. The ability of β-clamp to interact with many proteins at the same site makes it an important drug target, which was clearly validated for *M. tuberculosis* (Mtb)^17^. Knowledge derived from this study about the binding partners of β-clamp, the residues involved in these interactions, and their modes of interaction should help in understanding the underlying mechanism of replication and also in designing novel molecules that could potentially serve as drugs.

In this study, we determined the crystal structure of *H. pylori* β-clamp (Hpβ-clamp) followed by prediction of binding regions from ligase and pol III first by carrying out SPR experiments and then by Co-crystallizing Hpβ-clamp in complex with the peptide (clamp binding region) from HpDNA ligase. The region near BRCT domain was found to be the β-clamp interacting site. In addition, we used experimental studies to derive binding affinity measurements, and report for the first time in any organism an interaction between a β-clamp and DNAligase with a micromolar binding affinity. Also we report the similarities and differences between Hpβ-clamp structure and the structures of its homologs especially the differences in DNA binding loops.

## Results and Discussion

The genome of *H. pylori* rapidly evolves and shows wide geographical divergence. Since DNA replication and its control are central to bacterial proliferation, pathogenesis and virulence, we are here focusing on the β-clamp, also known as sliding clamp, which is a critical component of the DNA replication machinery and which serves as a processivity-promoting factor in DNA replication. Using STAMP^18^ we carried out structure-based multiple sequence alignment of Hpβ-clamp with its homologs (whose structures have been reported in the PDB) to find its closest homolog (Figure S1A). Furthermore, we carried out analysis in the Multiseq bioinformatics analysis environment^19^ in which we used STAMP structural alignment in order to construct a structure-based phylogenetic tree based on percent identity (Figure S1B). According to this tree, Hpβ-clamp has diverged considerably from its homologs.

### Basic architecture of β-clamp

**Figure 1:**
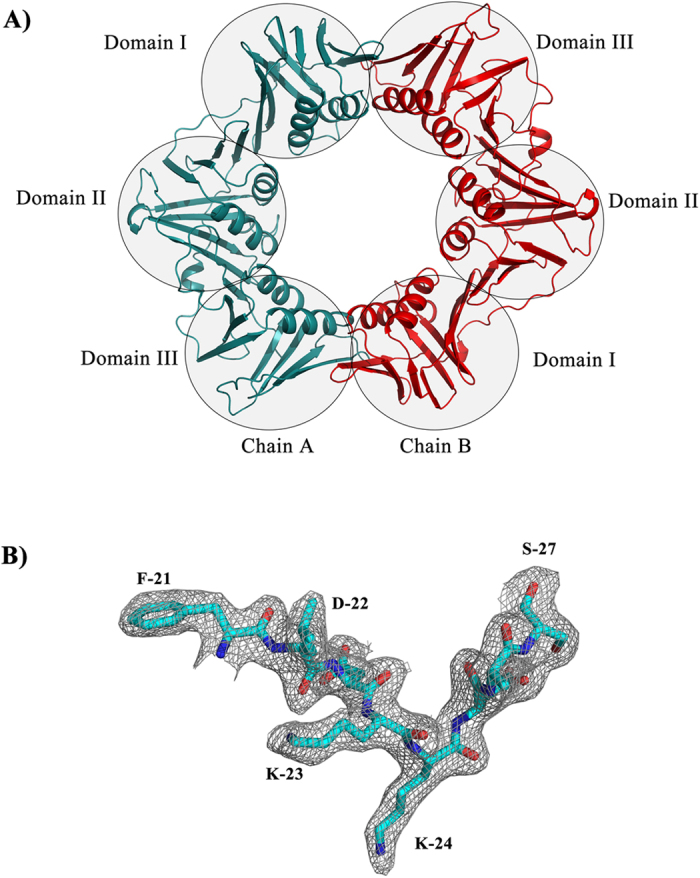
Crystal structure of Hpβ-clamp. (**A**) Cartoon representation of the crystal structure of Hpβ-clamp. Chain A is colored cyan and chain B is red. (**B**) 2Fo-Fc electron map at 1.0σ of Hpβ-clamp monomer.

In the space group C2, one monomer was observed in each asymmetric unit, which interacts with another monomer from asymmetric unit to form a dimer. In the space group P2 two monomers were present per asymmetric unit (Fig. 1A). However there is no significant difference observed between these two structures from different space groups, the structural alignment of both yielded an RMSD of 0.15 Å. While Hpβ-clamp was found to exist as both a monomer and dimer in the crystal, the dimer was the dominant component in solution according to a gel filtration experiment (Figure S2). The dimeric ring shaped structure is a biological functional unit that binds to DNA and slides along the DNA.

As expected for a prokaryotic β-clamp, each monomeric unit of Hpβ-clamp consists of three domains (Fig. 1A). In Hpβ-clamp, domains I, II, and III were observed to span residues Met1to Phe118, Pro119 to Pro 250, and Lys251 to Leu374, respectively. Domain I comprised of nine antiparallel β-strands and two alpha helices, domain II having eight β-strands and two alpha helices, and domain III with eight β-strands and two helices. In contrast, the first domain of *E. coli*β-clamp (Ecβ-clamp) was found to contain only eight β-strands^20^. Superimposition of all three domains of Hpβ-clamp (Figure S3) showed their similar topologies and secondary structures, except for few regions: the root mean square deviations (RMSDs) for 118 residues between domains I and II, for 131 residues between domains II and III, and for 123 residues between domains I and III were calculated to be 2.66 Å, 2.33 Å and 1.93 Å, respectively.

From the *E. coli* β-clamp co-crystal structure with DNA^21^ as well as from the structure with its interacting partners^22^,^23^,^24^,^25^, It is already known that domains I and II are involved in DNA binding, and domains II and III have the site for interaction with various β-clamp-binding proteins. A dual DNA- and protein-binding role for domain II is consistent with its good superposition with other two domains in our Hpβ-clamp structure. As in other clamps, twelve alpha helices were observed to form the inner surface of the Hpβ-clamp ring, with positively charged residues suitable for binding DNA. The β-sheets along with loops were found to form the outer surface of the ring.

### Dimeric interface residues

**Figure 2:**
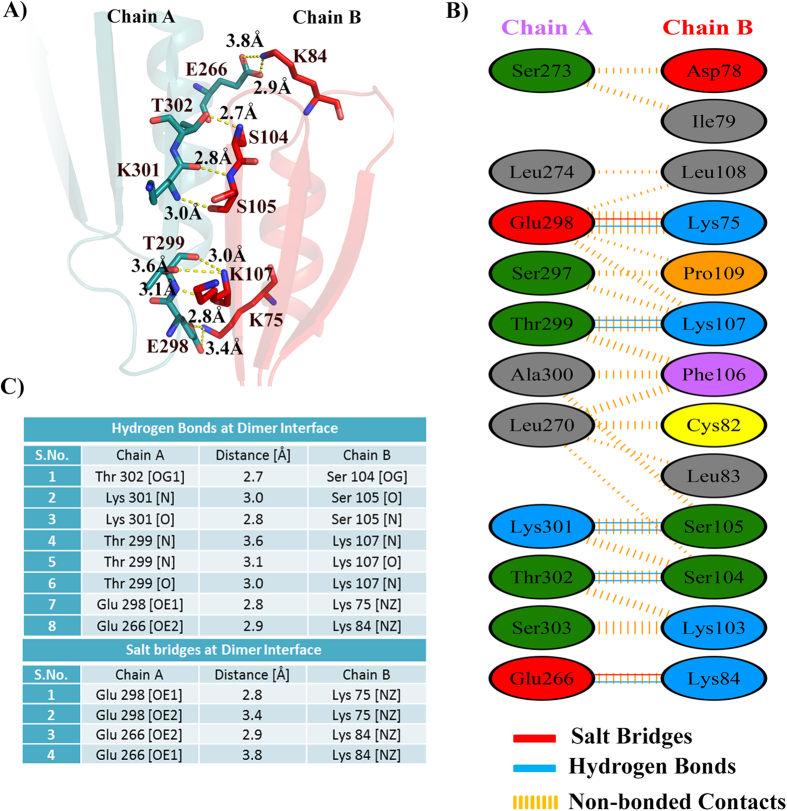
Residues present at dimeric interface. (**A**) Cartoon representation of dimeric interface between the two chains. Various ionic and hydrophobic interactions formed at the interface. Salt bridges formed between residue Glu266 of chain A and Lys83 of chain B and between Glu298 of chain A and Lys74 of chain B are shown in this figure. Another set of equivalent salt bridges also formed between Glu 266 of chain B and Lys 83 of chain A and between Glu 298 of chain B and Lys 74 of chain A in this homodimeric structure. (**B**) Pictorial representation of various residues involved in interaction between the two chains A,B. (**C**) Table describing the distance between the atoms interacting at dimeric interface.

As seen in β-clamp from other organisms, Hpβ-clamp form a head-to-tail dimer, i.e., the N-terminus of one monomer interacting with the C-terminus of another monomer. The residues interacting between chain A and chain B in the dimeric interface are shown in Fig. 2, Table S1. The several ionic and non-ionic interactions indicate a stable dimer, forming the ring structure. Two salt bridges are formed between Glu266 and Glu298 of chain A with Lys83 and Lys74 of chain B, and another set of equivalent salt bridges also formed between Glu 266 of chain B and Lys 83 of chain A, and between Glu 298 of chain B and Lys 74 of chain A. These salt bridges and several hydrophobic interactions were observed to mediate the association between the monomers. Overall, the observation of a relatively positively charged N-terminal domain and a negatively charged C-terminal domain (Figure S4) is consistent with ionic interactions dominating the dimerization of the *H. pylori* β-clamp.

### Comparison of the structure of Hpβ-clamp with those of other organisms

**Figure 3:**
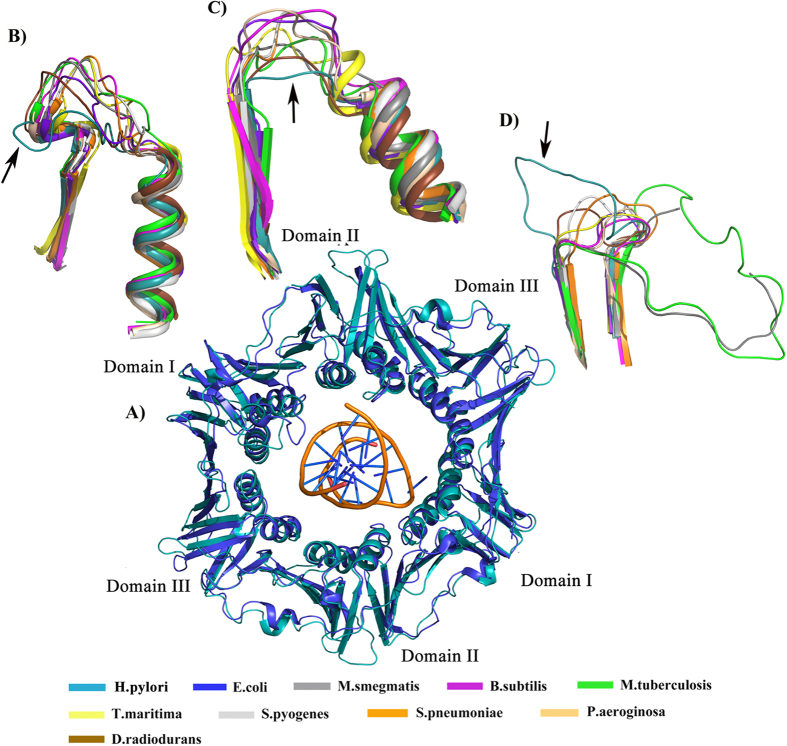
Structural alignment of Hpβ-clamp with DNA-bound Ecβ-clamp and other homologs. (**A**) Superimposition of Hpβ-clamp on DNA-bound Ecβ-clamp yielded an RMSD of 1.7 Å. The superimposition was done to analyze the Hpβ-clamp residues corresponding to Ecβclamp residues involved in DNA binding. (**B**) Magnified view of specific DNA-binding regions for the purpose of showing the differences between Hpβ-clamp and its homologs revealed the orientation of the domain I loop containing (in Hpβ-clamp numbering) residues 20–28 to be quite different in *H. pylori* than in any of its homologs, although with Lys23 of the Hpβ-clamp (corresponding to the DNA-binding Arg24 in *E. coli*) still in position to be close to DNA. (**C**) Magnified view of domain II loop containing (in Hpβ-clamp numbering) residues 210–214, which corresponds to the loop in Ecβ-clamp containing DNA-binding Asp208 and Gly209, revealed this loop to beuniquely short in Hpβ-clamp. (**D**) The β-clamp domain III loop containing residues 355–365 was observed to be larger in *H. pylori, M. tuberculosis* and *M.smegmatis* than in the other organisms.

Although superposition revealed the overall structure of Hpβ-clamp to be similar to the β-clamp structures in other organisms, but some important differences are there at the regions involved in DNA binding (Fig. 3). The electrostatic surface charge distributions of Hpβ-clamp and of all its homologs were calculated using the ABPS^5^ plugin in PyMOL (Figure S4). The N-terminal region of Hpβ-clamp was found to have more electropositive residues and the C-terminal region more electronegative residues at the dimeric interface. This distribution is similar to that in *E. coli* β-clamp, but different from that in *T.maritima* and *S.pyogenes* where the charges at the dimeric interface are evenly distributed.

#### DNA-binding region of Hpβ-clamp

A structural alignment between Hpβ-clamp and homologous β-clamps of other organisms showed an overall similarity in structure (Fig. 3A) but with some significant differences (Fig. 3B–D). The residues involved in DNA interactions in *E. coli* along with the corresponding residues in *H. pylori* and other organisms are given in Table S2. Perhaps most notably, two major DNA-interacting loops of β-clamp were observed to adopt quite different conformations in *H. pylori* compared to the β-clamp in *E. coli* and the other organisms.

The loop spanning residues 20 to 28 from domain I was found to be oriented in an opposite direction in the Hpβ-clamp structure compared to the corresponding loop in the clamp structures of other organisms (Fig. 3B). In the co-complex structure of *E. coli* β-clamp with DNA (PDB code: 3BEP), Arg24 interacts with DNA. Despite the oppositely oriented loops, our superposition of Hpβ-clamp with DNA-bound Ecβ-clamp showed the side chain of Hpβ-clamp Lys23 (which corresponds to Arg 24 Ecβ-clamp) still to be in a position close to DNA, suggesting that Lys23 of Hpβ-clamp may participate in DNA binding and may be crucial for binding to DNA. The average B-factor for this region was calculated to be greater for *E. coli* (with and without DNA) and other organisms as compared to *H. pylori* (Table S3) indicates that this loop is relatively rigid in Hpβ-clamp, but flexible in the β-clamps of the other organisms.

In domain II, the loop containing residues 210–214 was observed to be shorter in Hpβ-clamp than in *E. coli* and the other homologs (Fig. 3C). The residues Asp208 and Gly209 in the corresponding loop in *E. coli* are involved in DNA interactions. Moreover, in *E. coli* and the other homologs, the B-factor for this region was found to be quite high, compared to that for the other regions in the protein, in contrast to the case in *H. pylori*. These results indicate that this loop in other organism may interact with DNA, however this shortened loop in *H. pylori* may not be involved in DNA interactions because of the absence of certain residues.

In addition to these two loops, another difference in the Hpβ-clamp was observed at the central channel of the clamp structure near the DNA-binding site in the loop encompassing residues 355–365 (Fig. 3D). This loop was observed to be much larger in the clamps of *H. pylori, M. tuberculosis and M. smegmatis* than in *E. coli* and other homologs. Moreover in case of *E. coli* the average B-factor for this loop was found to be near the mean B value but in *H. pylori*, *M. tuberculosis and M.smegmatis,* it was found to be higher when compared to their mean B values. This indicates the flexibility of this loop in *H. pylori* and these other two organisms. Also, In *H. pylori*, this loop is lined with basic residues K357, H360, and K364. Furthermore, the significance of this loop is supported by its presence, as large loops, in both mycobacterium species. These observations suggest that loop 355–365 should be involved in Hpβ-clamp-DNA interactions, even though the corresponding region in the Ecβ-clamp is not involved in binding DNA.

### Search for proteins that bind Hpβ-clamp

Co-crystal structures of Ecβ-clamp with peptides from Pol II (PDB ID: 3D1E)^4^, the Pol III alpha subunit (PDB ID: 3D1F)^4^, and Pol V (PDB ID: 4K74)^22^ as well as with short protein regions of the Pol III delta subunit (PDB ID: 1JQL)^23^ and Pol IV (PDB ID: 1UNN)^24^ have been deposited in the PDB. Many proteins involved in replication initiation that bind β-clamp have been identified/annotated in *E. coli* but not yet in *H. pylori*. We searched the NCBI database and found that the alpha and delta subunits of DNA pol III and DNA ligase are well annotated in *H. pylori*. Based on these findings, we searched for β-clamp-binding regions in the Pol III alpha subunit and in DNA ligase.

#### Alpha subunit of DNA pol III in *H. pylori*

In *E. coli*, out of its several β-clamp-interacting partners, one important such partner is the alpha subunit of DNA polymerase III, which catalyzes the polymerase activity of the holoenzyme complex^26^. Here a bipartite association of β-clamp and alpha subunit of DNA pol III exists. There are two binding sites in this alpha subunit in *E. coli*^27^: one is near the C-terminus (residues 1154–1159 (QVELEF)) and the other is in the middle of the subunit (residues 920–924 (QADMF))^27^. Mutation in any binding site affected the chromosomal replication. Since these sites are essential for polymerase activity, they are of much interest. In our study, we identified one of these sites in the *H. pylori* alpha subunit of DNA Pol III. We predicted that Hpβ-clamp interacts with the DNA Pol III alpha subunit with the sequence 953QGGNSLF959, which is close to the C-terminal region of this subunit.

#### *H. pylori* DNA ligase has a β-clamp binding site

Apart from the alpha subunit of DNA Pol III, DNA ligase is also an interesting binding partner of sliding clamps. In eukaryotes (in particular, *Sulfolobus solfataricus*), the interaction between PCNA and DNA ligase was well studied^28^. In the case of prokaryotes, the interaction between β-clamp and DNA ligase has not been studied in detail. Lopez de Saro and O’Donnell^10^ examined Ecβ-clamp for its interaction with EcDNA ligase by performing a native gel mobility shift assay. They have also identified MutL & MutS will also bind to β-clamp in *E. coli*. However Vandana Kukshal *et al.*^11^ reported that *M. tuberculosis* DNA ligase do not interact with β-clamp and they hypothesized that some other factor may be involved that aids the interaction between these two proteins. Such findings made us curious to determine whether an interaction between HpDNA ligase and Hpβ-clamp exists or some other factor is involved that mediates the interaction between these two proteins. Based on the current understanding of β-clamp binding motifs^7^,^29^, we predict the _554_QEFIRSLF_561_ sequence near the C-terminal BRCT domain of *H. pylori* ligase to be an Hpβ-clamp-binding site. Note that a β-clamp-interacting site in ligase has not been previously reported in any prokaryotic organism.

### Docking studies suggest that ligase and the α-subunit of Pol III can bind Hpβ-clamp

To evaluate the interactions between Hpβ-clamp and each of the two sites described above, i.e., one from the *H. pylori* DNA Pol III alpha subunit and the one from *H. pylori* DNA ligase, docking studies of Hpβ-clamp to short peptides from each of these two sites were performed. Information regarding the β-clamp binding site was deciphered from the co-crystal structure of *E. coli* β-clamp with the β-clamp-binding peptide from *E. coli* Pol III alpha subunit (PDB id 3D1F)^4^. The binding energy values calculated for Hpβ-clamp with the peptide from the DNA Pol III alpha subunit as well as DNA ligase were comparable to the binding energy for Ecβ-clamp with the peptide from its Pol III alpha subunit (Table S4).

### Confirmation of the interactions between Hpβ-clamp and Hpβ-clamp-binding regions in ligase/α-subunit of Pol III by SPR studies

**Figure 4:**
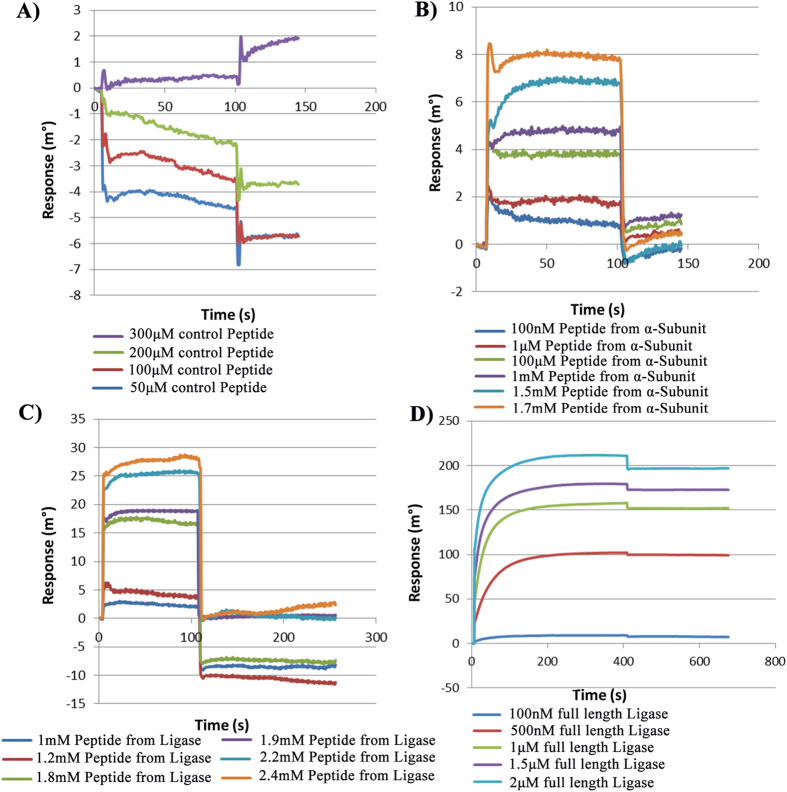
SPR sensograms measuring the interaction of Hpβ-clamp (ligand) with peptides (analytes) from its interacting partners and with full-length HpDNA ligase. The Hpβ-clamp-binding region in the alpha subunit of DNA Pol III and DNA ligase were docked and peptides from those regions were generated and in different concentrations passed over the β-clamp that was immobilized on the chip. Since peptides are very small moeities and our Autolab SPR is not very sensitive to such experiments, the signals generated were very weak. The association phase lasted upto 100 seconds, followed the the onset of dissociation. (**A**) A random peptide (EQDSLFGG) was taken as a control. (**B**) Interaction study with a peptide from the C-terminal region of the alpha subunit of DNA PolIII with concentrations ranging from 100 μM to 1.7 mM. (**C**) Interaction study of a peptide from HpDNA ligase at concentrations ranging from 1 mM to 2.4 mM. (**D**) Interaction study of β-clamp with full-length DNA ligase ranging from 100 nM to 2 μM. The affinity of ligase for β-clamp was calculated to be 1.25 μM.

We further experimentally confirmed the binding of these regions to Hpβ-clamp through SPR by immobilizing Hpβ-clamp on the surface of the sensor chip and passing different concentration of the peptides over the chip surface (Fig. 4). The peptide from alpha showed the binding affinity in millimolar range. To further confirm the molecular interaction between Hpβ-clamp and Hpligase, SPR-based interaction studies were performed using Hpβ-clamp as the ligand and HpDNA ligase as the analyte. The interaction between the two was found to be in the micromolar range: specifically, the affinity of HpDNA ligase for Hpβ-clamp was calculated to be 1.25 μM (Fig. 4D). We also checked interaction between Hpβ-clamp and HpDNA ligase using SAXS experiment (Figure S5). The radius of gyration (Rg) for β-clamp and ligase alone was calculated to be 3.87 with a Dmax 9.8 nM and 4.73 with a Dmax 14.5 nM respectively. However Rg for the mixture of β-clamp and ligase was calculated as 6.3 with a Dmax 22 nM which is just suggesting the formation of complex.

### Confirmation of peptide_554_QEFIRSLF_561_ from ligase as Hpβ-clamp binding region through co-crystallization

**Figure 5:**
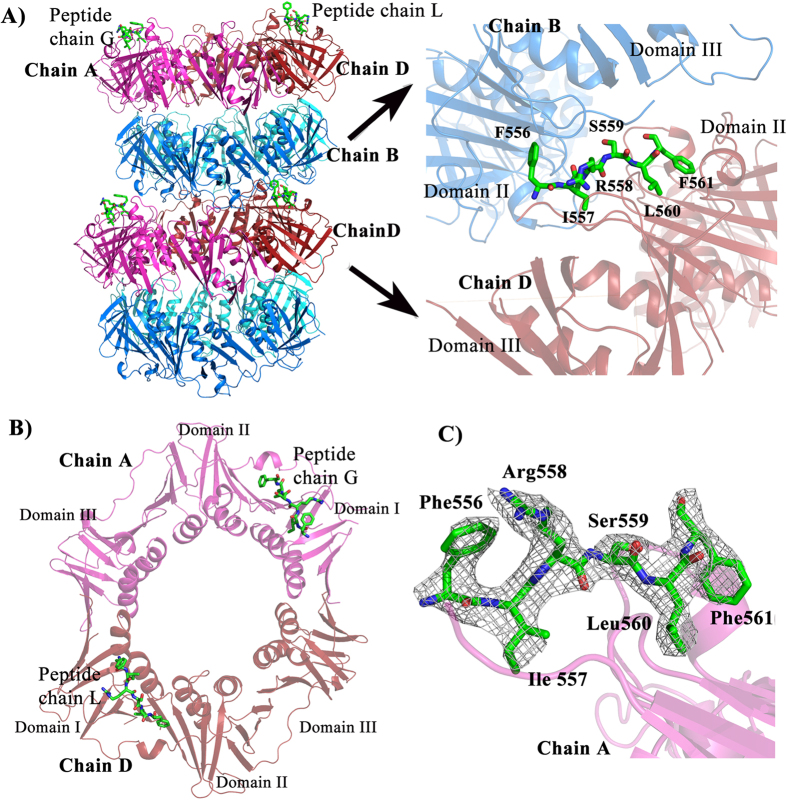
Crystal structure of Hpβ-clamp bound to a peptide from HpDNA ligase. (**A**) Cartoon representation of two adjacent asymmetric units in ligase peptide bound β-clamp. The arrangement of molecules is in such a manner that peptide bound to chain D also interacts with chain B. However no such interaction exists for the other peptide (chain G) bound to chain A. The enlarged view of the peptide bound to the conserved binding pocket of the beta clamp in such a manner that it seems to be present at the interface between two asymmetric units, where Phe556 interacts with different subunit (i.e., between chain B of one asymmetric unit and chain D of another asymmetric unit). (**B**) Cartoon representation of β-clamp dimer showing the location of bound peptide. The peptide bound to the conserved binding pocked located in between Domain II and Domain III. (**C**) 2Fo-Fc electron density map at 1.0σ of peptide from HpDNA ligase bound Hpβ-clamp. The peptide whose electron density is shown here is bound to chain A. Electron density for peptide (FIRSLF) is seen, however for terminal residues (Q554 and E555) electron density is missing.

**Figure 6:**
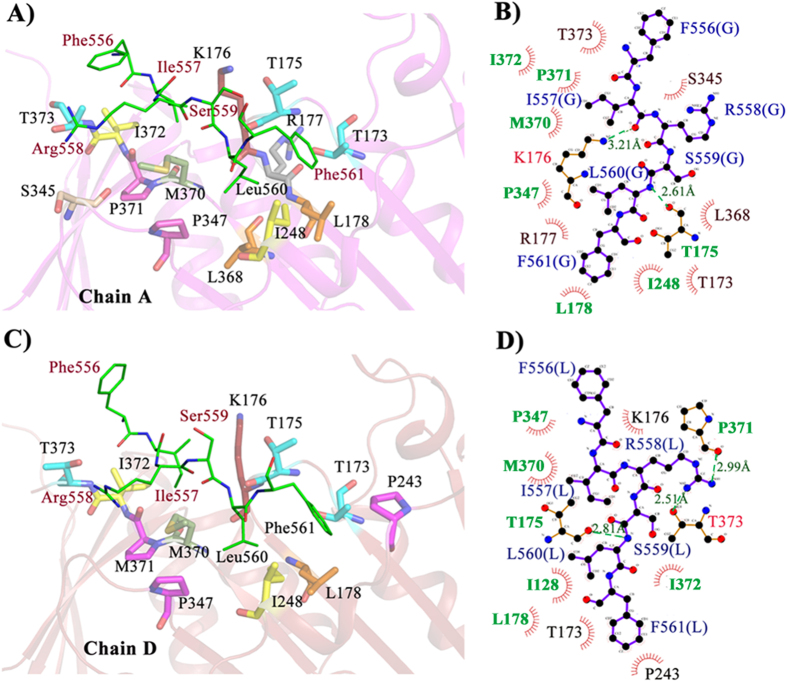
Interaction between the ligase peptide and residues in the Hpβ-clamp protein-binding pocket. (**A**) Cartoon representation of the protein-binding pocket of beta clamp chain A. Interacting residues of β-clamp are shown in a stick model. (**B**) Ligplot of peptide-bound beta clamp chain A. The binding of peptide to the beta clamp pocket was dominated by hydrophobic interactions along with two hydrogen bonds formed by Thr175 and Lys176 of β-clamp with Leu560 and Ile557 of the peptide. (**C**) Cartoon representation of the protein-binding pocket of beta clamp chain D. Interacting β-clamp residues are represented by sticks. (**D**) Ligplot of peptide-bound β-clamp chain D. All residues of β-clamp showed here form hydrophobic interactions with the peptide. Both residues Phe371 and Thr373 of β-clamp form hydrogen bond with Arg558 of the peptide; T175 of β-clamp form hydrogen bond with Leu560. The residues which are conserved in both the peptide chains G and L are colored green.

To validate the region in HpDNA ligase predicted by us to be an Hpβ-clamp binding region, we co-crystallized Hpβ-clamp with the peptide _554_QEFIRSLF_561_ from DNA ligase. The co-crystal structure belongs to space group P2_1_2_1_2_1_ with four monomers making two dimers (first above the second) per asymmetric unit (Fig. 5A). Out of two dimers, the peptide was found to occupy both the binding sites of the first dimer (Fig. 5B), while second clamp dimer did not show any peptide bound to them. Interestingly, the arrangement of molecules in the crystal is in such a way that one of the peptide (chain L) observed in between two chains (chain D & chain B) of two adjacent asymmetric units, interacting with both the chains. However no such contact was found for the other peptide (chain G) occupying the other side of dimer i.e the peptide seen interacting with only one β-clamp chain A. In other words, one peptide (Chain L) bound to two molecules of β-clamp (Chain B & D), while another peptide (Chain G) bound to single β-clamp (Chain A). This kind of symmetry related interactions must be crystal artifact and should not have any functional significance, while common mode of binding in both chains (Chain A and D) should be related to functional significance. The 2Fo-Fc electron density map (Fig. 5C) for bound peptide to Hpβ-clamp and temperature factors of the peptide are comparable to β-clamp indicating strong binding. The binding of the peptide to the binding pocket of beta clamp is dominated by hydrophobic interactions. Out of eight residues of peptide _554_QEFIRSLF_561,_ the density for Gln554 and Glu555 was missing in both the chains. Ile557, Leu560 and Phe561 interact with the residues that are present in the cleft between domain II and III in both the chains (Fig. 6) suggesting their importance for β-clamp interaction. Arg558 of chain L is making hydrogen bond with residue P371 and T373 of Chain D. Chain A is oriented little differently, K176 and T175 makes hydrogen bond with I557 and L560 of peptide chain L respectively. In total the residues Thr173, Thr175, Lys 176, Leu178, Ile248, Pro347, Met 370, Pro371, Ile372, Thr373 from chain A and chain D of β-clamp are involved in interaction with both peptide chains (chain G and chain L) almost in similar manner.

A structural alignment of native β-clamp structure with chain A and chain D of the β-clamp complex structure yielded RMSDs of 1.17 and 1.07, which suggests little movement of this region upon peptide binding. A comparison of the native β-clamp structure with the peptide-bound β-clamp structure showed differences in the positions of some of the residues at the protein-binding pocket of β-clamp (Figure S6). The residues involved in peptide binding reveal the maximal local displacement, the positions of residues Lys176 and M370 of β-clamp were found to differ between these two structures compared to native structure. In peptide bound structure, both of them get oriented in opposite directions as compared to their original orientation in native structure. The residues T173, T175, I178, I248, M370, P371, I372 and T373 in binding pocket shifts from their native positions so as to create an absolute cleft. Such a movement of these residues helps in forming a continuous stretch of a cleft that is receptive to the binding of an interacting partner. One noticeable thing is that residue Thr175 of β-clamp in both the chains makes hydrogen bond with backbone nitrogen of Leu560 from the peptide, suggesting that this must be a conserved residue in the protein-binding pocket.

**Figure 7:**
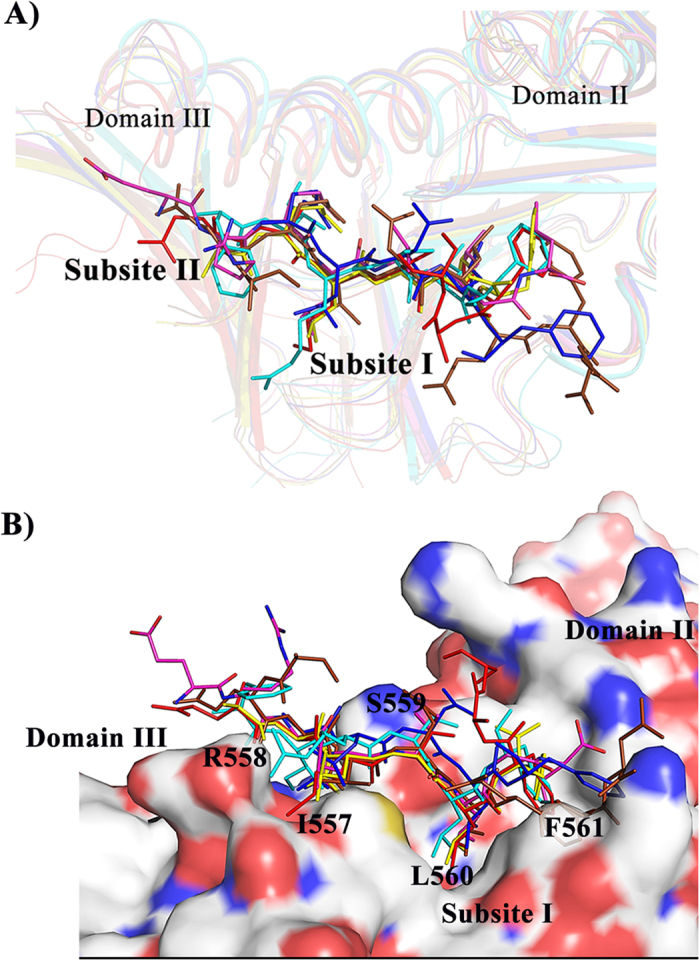
Structural alignment of Hpβ-clamp bound ligase peptide with different peptide bound *E. coli* β-clamp structures. (**A**) The structural comparison of *E. coli* β-clamp peptide bound structures with Hpβ-clamp ligase bound structure shows β-clamp interacting partners interacts in very similar manner. Most of the interacting residues in β-clamp are conserved. The color code for various structure used for this alignment is as follows: *H. pylori* β-clamp complex with Peptide from DNA ligase (FIRSLF) (cyan), *E. coli* β-clamp complex with delta subunit (QAMSLF) (red), *E. coli* β-clamp complex with pol IV (ERQLVLGL) (Pink), *E. coli* β-clamp complex with peptide from alpha subunit of DNA pol III (SEQVELEFD) (brown), *E. coli* β-clamp complex with peptide from Pol V (QLNLF) (Blue), *E. coli* β-clamp complex with peptide from pol II (QGLGLF) (yellow). (**B**) Surface representation of the β-clamp protein binding cleft. The last five residues of the peptide (IRSLF) in HpDNA ligase and the corresponding residues in *E. coli* binding partners are mainly responsible for binding.

The comparison of the protein-binding region of Hpβ-clamp with the protein-binding regions of other homologs shows some differences in the loops at the protein-binding site, but binding is seen at similar groove between domains II and III (Figure S7). The comparison of peptide bound structures of *E. coli* [Peptides from *E. coli*’s DNA pol. II (PDB id: 3D1E), delta subunit of DNA pol. III (PDB id: 1JQL), alpha subunit of DNA pol. III (PDB id: 3D1F), little finger domain of DNA pol. IV (PDB id: 1UNN) and pol.V (PDB id: 4K74)] with ligase peptide bound *H. pylori* β-clamp structure shows that the binding pattern of β-clamp interacting proteins is same with minute differences (Fig. 7) and indicates the conserved mode of binding of β-clamp binding proteins. All the peptide interactions are dominated by hydrophobic residues, especially that binds to subsite I.

The peptide _554_QEFIRSLF_561_is located near the C-terminal BRCT domain of ligase. Note that BRCT stands for the breast cancer susceptibility protein C-terminal domain, and this domain is located at the C-terminal portion of the BRCA-1 protein, whose gene is on the long arm of chromosome 17. Most of the proteins in this BRCT family are involved in cell-cycle regulation or in DNA repair, suggesting a role for these BRCT domains in these pathways. Also, these domains are thought to function as a protein-protein interacting moiety. Based on the results of peptide docking, peptide SPR, full-length ligase-Hpβ-clamp SPR, and the co-crystal structure, the region near the C-terminal region of the BRCT domain, i.e., that containing QEFIRSLF appears to be involved in binding Hpβ-clamp. Further in future, the importance of these peptides could be deduced by performing functional assay, mutational analysis including the study of their full protein complex (in solution using SAXS or EM) or SPR interaction studies.

## Conclusion

The overall structures of all β-clamp proteins are very similar, but the proteins with which they interact differ. It is known that ligase from *E. coli* interacts with its β-clamp, while in Mtb, β-clamp and ligase do not interact. The ability of *H. pylori* to undergo mutations and recombination at a high rate makes it different from other organisms. Nevertheless, despite the differences between the protein-protein interaction sites of *H. pylori* and *E. coli,* in both organisms β-clamp and ligase interact with each other, and as determined by us the region of ligase involved in binding β-clamp is located near the C-terminal BRCT domain. The binding of ligase peptide at both interacting sites indicates that both sites of Hpβ-clamp could be used simultaneously as a processivity promoting factor. Also comparison of Hpβ-clamp with its homologs showed clear differences at the DNA-binding loops and differences in the details of the peptide-binding regions. These observations suggest that the pattern in which Hpβ-clamp interacts with DNA may not be exactly the same as seen in *E. coli*, except for some conserved residues. The protein-binding site on β-clamp, which recruits several proteins including ligase, is a very important drug target^17^, and the current crystal structure makes it possible to use this site to rationally design an *H. pylori*-specific drug. From the complex structure, we could propose much smaller signature sequence (ZXSLF) for Hpβ-clamp binding site, where X represents any hydrophilic residue; X represents any small hydrophobic amino acid, although it needs further validation. Also the elucidation of this complex structure is an initiative step towards enlightenment in the ligase-β-clamp interaction in eubacteria and further will help us in understanding the mechanism during replication.

## Materials and Methods

### Cloning, overexpression and purification of β-clamp

The gene sequence (1122 bp) of β-clamp from *Helicobacter pylori* 26695 was chosen from the NCBI database and it was PCR amplified using the forward oligo 5′ CATGCCATGGCATGAAAATCAGTGTTAGTAAAAACGA 3′ and the reverse oligo 5′ CGGCTCGAGTAGTGTGATTGGCATCATCAAA 3′. The PCR-amplified products were digested using the restriction enzymes NCoI and XhoI at 37 °C. Overnight ligation into the pET 28(b) vector was done at 16 °C. The ligated product was transformed into the *E. coli* DH5-α strain. The clone was confirmed by colony PCR followed by plasmid PCR (Figure S2A), and the recombinant plasmid was transformed into the *E. coli* BL21 strain.

For overexpression, the cells were initially grown at 37 °C, and, as they reached an OD of 0.6, expression was induced by adding IPTG to reach final concentration of 0.5 mM. The cells were incubated at 30 °C for five hours in a shaker, and after incubation the culture was harvested by centrifugation at 6000 rpm for ten minutes at a temperature of 4 °C. The cells were suspended in suspension buffer containing 30 mM Tris-HCl pH 7.5, 150 mM NaCl, 10 mM imidazole, 6 mM beta-mercaptoethanol, 0.5%Tween 20. To lyse the cells, 0.3 mg/ml of lysozyme was added and incubated at 4 °C for 30 minutes; the resulting soup was later sonicated and then centrifuged at 13000 rpm for thirty minutes.

The clear supernatant derived from the centrifugation of the lysed cells was applied to a Ni Sepharose column that had been pre-equilibrated with 30 ml of buffer containing 30 mM Tris pH 7.5, 150 mM NaCl, 6 mM βMe and 10 mM imidazole. After several washes with low (5–30 mM) concentrations of imidazole, the protein was eluted in buffer containing 50 mM Tris pH 7.5, 150 mM NaCl, 150 mM imidazole, 30 mM arginine and 6 mM βMe. The eluted fraction was concentrated using a Centricon filter (from Millipore). For further purification and removal of imidazole, the concentrated protein was loaded into a HiLoad G200 16/60 column and eluted with a flow rate of 1 ml/min (Figure S2C). The column was pre-equilibrated with the running buffer having 30 mM Tris pH7.5, 150 mM NaCl, 5% glycerol, 30 mM arginine and 6 mM βMe. The purified fractions were pooled and concentrated by using a centricon. Tryptic digestion and mass fingerprinting using a Bruker ESI-ion trap mass spectrophotometer were carried out to confirm that the desired protein was obtained. The protein concentration was determined by measuring its UV absorption at 280 nm. An approximate extinction coefficient of 10680 M^−1^ cm^−1^ for β-clamp was calculated using the ExPASy server^30^, and used for this protein concentration determination.

### Cloning, overexpression and purification of NAD-dependent DNA ligase

The gene sequence of NAD-dependent DNA ligase from *Helicobacter pylori* 26695 was also chosen from the NCBI database, using a procedure similar to that used for Hpβ-clamp. The ligase sequence was PCR amplified using the forward oligo 5′ CATGCCATGGGCATGATAAAAAGCCAAAAAGAATATTTAGAAAG 3′ and the reverse oligo 5′ CCGCTCGAGATCCAATTCTTTAAGGCGCTTTAATAAT 3′. The cloning and expression of ligase was performed in a similar manner to that forβ-clamp (Figure S8). A Ni Sepharose column was pre-equilibrated with buffer having 30 mM Tris pH 8.5, 150 mM NaCl, 6 mM βMe and 10 mM imidazole and loaded with the clear supernatant. After several washes with various concentrations of imidazole, the protein was eluted in a buffer containing 50 mM Tris pH 8.5, 150 mM NaCl, 30 mM imidazole, 50 mM arginine and 6 mM βMe. The eluted fraction was concentrated using a Centricon. The protein was further purified with a HiLoad G200 16/60 column at a flow rate of 1 ml/min (Figure S8C). Arginine and glycerol were added to the gel filtration buffer to increase the solubility and stability of the protein. The purity of the desired protein was checked using 12% SDS-PAGE. The purified fractions were pooled and concentrated by using a Centricon. Protein concentration was determined by measuring UV absorption at 280 nm. An approximate extinction coefficient of 53790 M^−1^ cm^−1^ for ligase was calculated using the ExPASy server and used for this protein concentration determination.

### Crystallization and X-ray data collection of native Hpβ-clamp and that in complex with an HpDNA ligase peptide

Purified β-clamp protein was concentrated up to 5 mg/ml in 30 mM Tris pH 7.5 buffer also containing 150 mM NaCl, 6 mM beta-mercaptoethanol, 5% glycerol and 30 mM arginine. Extensive robotic (MOSQUITO) crystallization trials using the hanging-drop vapour-diffusion method and using the Morpheus Screen^31^ yielded cube-shaped crystals. Each 600 nl drop was set in a 1:1 ratio of protein and precipitant, and equilibrated against 200 μl of precipitant. Crystals appeared within 5 days from a solution containing 10% w/v PEG 2000, 20% v/v PEG MME 550, 0.03 M divalent cation (MgCl_2_ and CaCl_2_), and 0.1 M MOPS/HEPES-Na pH7.5. The condition was refined, and high-quality crystals were obtained from a solution containing 6% w/v PEG 20,000, 20% v/v PEG MME 550, 0.01 M MgCl_2_, 0.1 M MOPS/HEPES-Na pH7.3, and 0.2 M ammonium citrate. The crystals were equilibrated with a cryoprotectant solution containing 5% w/v PEG 20,000, 25% v/v PEG MME 550, 0.2 M ammonium citrate, and 0.1 M HEPES/MOPS pH 7.3, and were then mounted in cryoloops and flash frozen in liquid nitrogen at 100 K. The crystals diffracted X-rays to a resolution of 1.8 Å–2.0 Å.

For co-crystallization, the native Hpβ-clamp protein was incubated overnight with a ligase peptide in 1:2 ratios and the resulting cocktail was used for co-crystallization using similar conditions used for native Hpβ-clamp protein. Needle-shaped crystals were obtained within two days. The cryoprotectant used was the same as that used for native crystals. X-ray diffraction data from these crystals were collected upto a resolution of 2.9 Å.

Data for all the crystals were collected at DBT-ESRF beam line BM14. The data were processed and integrated using HKL 2000.

### Structure solution and refinement

The crystal structure of Hpβ-clamp was solved by molecular replacement using the crystal structure of β-clamp from *Pseudomonas aeroginosa* (pdb code: 4TR8) as a search model, which showed 46% sequence similarity and 94% query coverage with Hpβ-clamp. Phaser 2.3.0^32^ was used for molecular replacement. The solution was obtained in the space group C2 (PDB code: 4RKI) with one chain per asymmetric unit, and in the space group P2 (PDB code: 4S3I) with two chains per asymmetric unit. For the space group C2, the model resulting from molecular replacement showed an R-factor and Rfree of 44% and 50%, respectively. Multiple rounds of manual adjustment of the model in COOT^33^ and refinement with Refmac5^34^ were carried out to 2.1-Å resolution, which brought the R/Rfree value down to 21/25and resulted in unambiguous electron density. Finally, the stereochemistry of the model was evaluated by applying PROCHECK, and these values as well as the refinement statistics are listed in Table 1. The structure of the P2 crystal was solved based on the Hpβ-clamp monomer from the space group C2. The data for the β-clamp-ligase peptide complex was solved by molecular replacement using the native crystal structure with space group C2. The structure of the complex was solved in the space group P2_1_2_1_2_1_ (PDB code: 5frq) with four chains (making two rings) per asymmetric unit. Some regions of the complex structure, mainly the loop regions had poor electron density and not refined well, which resulted some residues in disallowed regions of Ramachandran plot.

### Search for β-clamp-interacting partners

Being a processivity-promoting factor for a number of enzymes, β-clamp interacts with many proteins. β-clamp in *E. coli* (Ecβ-clamp) has been found to interact with MutS, DNA ligase, DNA polymerase I, the UmuC subunit of DNA polymerase V, the δ subunit of DNA polymerase III, DNA pol IV, Hda, and the α subunit of DNA pol III^10^,^22^,^23^,^24^,^25^,^35^. Co-crystal structures of Ecβ-clamp with its interacting partners have been reported and deposited in the Protein Data Bank (PDB). Based on the analysis of β-clamp binding binding motifs, we analysed and predicted β-clamp interacting partners, namely α subunit of DNA Pol III and NAD-dependent DNA ligase, in *H. pylori.* One interaction site from the α subunit of DNA pol III, whose sequence is QGGNSLF, and one from NAD-dependent DNA ligase, i.e., QEFIRSLF, were predicted to interact with Hpβ-clamp based on their sequence conservation. These peptides were then subjected to molecular docking simulations against the Hpβ-clamp protein-binding site using the Schrodinger Glide program^36^,^37^. The docking scores along with binding energy values are listed in Table S1.

### Structural analysis

Coordinates of the β-clamp structures from different organisms used for comparison were obtained from the PDB. These structures were from *E. coli* (PDB code: 1MMI, 3BEP)^38^, *Thermotoga maritima* (PDB code: 1VPK), *Streptococcus pyogenes* (PDB code: 2AVT)^39^, *Streptococcus pneumonia* (PDB code: 2AWA), *Mycobacterium tuberculosis* (PDB code: 3RB9)^11^, *Mycobacterium smegmatis* (PDB code: 5AH2)^17^, *Bacillus subtilis*(PDB code: 4TR6)^6^, *Pseudomonas aeruginosa* (PDB code: 4TR8)^6^ and *Deinococcus radiodurans* (PDB code: 4TRT)^40^. PromalS3D was used to create the structure-based multiple sequence alignment. Interactions among dimeric interface residues and protein-peptide residues were calculated using the program Contact in the CCP4 suite^41^. A structure alignment was produced by using RAPIDO^42^,^43^. The sequence similarities (%) and structural similarities (RMSD) are given in Table S5.

### Surface plasmon resonance of β-clamp with full-length NAD-dependent DNA ligase, peptides from DNA ligase, and α subunits of DNA pol III

In order to determine the strength of the interaction of β-clamp with the peptide from ligase (_**554**_**QEFIRSLF**_**561**_), the α-subunit of DNA pol III, and full-length ligase, surface plasmon resonance (SPR) experiment was carried out using Autolab SPR (at Advanced Instrumentation Research Facility, Jawaharlal Nehru University). The surface of the sensor (self-assembled monolayer of 11-mercaptoundecanoic acid (MUA) on a gold surface) was activated with N-hydroxysuccinimide (NHS, 0.05 M)/N-ethyl-N-(diethylaminopropyl) and carbodiimide (EDC; 0.2 M). β-clamp at a concentration of 50 ng/ml was immobilized to the activated sensor surface in filtered and degassed 10 mM sodium acetate buffer at pH 5.0. Two channels were used for the experiment, the first was used for the test and the second was taken as a control. Hpβ-clamp ligand was immobilized in both channels, but the analyte (one of the β-clamp-binding peptides or proteins) was only passed through the first channel while only buffer was passed through the second channel. After ligand immobilization, the surface was blocked with 100 mM ethanolamine at pH 7.4 and regeneration was done using 50 mM NaOH. In running buffer containing 10 mM HEPES [pH 7.4], 150 mM NaCl, 3 mM EDTA, and 0.05% NP-40 surfactant, the association kinetics for β-clamp was monitored for 400 s, followed by monitoring the dissociation for 300 s. Different concentrations of peptides from DNA ligase and of the α subunit of DNA pol III were prepared in running buffer and injected across the sensor surface. SPR signals and resultant sensograms for β-clam p-peptide interactions were analyzed. Similarly, different concentrations of DNA ligase, including 100 nm, 500 nM, 1 μM, 1.5 μM, and 2 μM, were passed across the sensor surface. Changes in the signals on the activated/blocked control panel were subtracted from those resulting from the binding of DNA ligase to β-clamp using an in-line reference signal, and the resultant sensogram was analyzed. Finally the surface was regenerated with 75 mM NaOH. All of the data were recorded at room temperature (i.e., 25 °C). The data analysis was performed using Autolab SPR Kinetic Evaluation software.

## Additional Information

**How to cite this article**: Pandey, P. *et al.* Structural insight into β-Clamp and its interaction with DNA Ligase in *Helicobacter pylori. Sci. Rep.* **6**, 31181; doi: 10.1038/srep31181 (2016).

## Supplementary Material

Supplementary Information

## Figures and Tables

**Table 1:** Crystallographic data and refinement statics of *H. pylori* beta-clamp.

Data collection	C2	P2	β-clamp with ligase peptide
Space group			P2_1_2_1_2_1_
Cell parameters			
a,b,c (Å)	89.5, 65.8, 82.7	82.5, 65.4, 88.9	64.8, 146.1, 179
α,β,γ (deg.)	90.0, 115.7, 90.0	90.0, 116.0, 90.0	90, 90, 90
X-ray source	BM14, ESRF, France	BM14, ESRF, France	BM14, ESRF, France
Wavelength (Å)	0.769	0.976	0.953
Molecules in Asymmetric unit	One monomer	One dimer	Two Dimer
R_sym_ or R_merge_ (%)	7.1 (47.4)	6.2 (49)	9.5 (51.5)
Number of observations			
Unique	52832	60966	35997
Total	203346	619736	152232
Completeness (%)	98.56 (95)	100 (100)	92.9 (95)
Mean I/σ	29.4(2.05)	38.35 (2.8)	11 (1.9)
Refinement			
Resolution range (Å)	50–2.05	80.0–1.95	50–2.9
Rwork/Rfree	21.4/24.7	21.4/25.0	23.1/26.1
Number of atoms			
Protein	2967	5944	11503
Water	80	391	10
r.m.s deviation			
Bond angles (deg.)	1.136	1.114	1.793
Bond lengths (Å)	0.006	0.0008	0.013
Mean B value	35.628	27.870	26.33
Ramachandran plot			
Most favoured regions (%)	311 (89.4)	636 (91.4)	1350 (95.3)
Generously allowed regions (%)	1 (0.3)	6 (0.9)	35(2.5)
Disallowed regions (%)	1 (0.3)	3(0.4)	32(2.3)
