# Supplementary material for: Structural insight into β-Clamp and its interaction with DNA Ligase in Helicobacter pylori
Source: Sci Rep. 2016 Aug 8;6:31181. doi: 10.1038/srep31181 (PMC4976356; doi:10.1038/srep31181)
Supplement: Supplementary Information [file srep31181-s1.pdf]

Structural insight into β-Clamp and its interaction with DNA Ligase in *Helicobacter pylori*

Preeti Pandey[a,b], Khaza Faisal Tarique[a], Mohit Mazumder[a], Syed Arif Abdul Rehman[a], Nilima kumari[b] and Samudrala Gourinath[a*]

Supplementary Figures

Figure S.1 Structural analysis of Hpβ-clamp and its homologs. A) Structure-based sequence alignment of Hpβ-clamp (whose crystal structure we determined here) with its homologs. The solvent accessibility values of the residues in Hpβ-clamp are rendered by the bar below the sequences: blue, cyan, and white denote highly accessible, intermediately accessible and buried residues, respectively. In the sequence block, the residues colored white on a red background are completely conserved in the sequences shown, and the residues colored red on a white background are partially conserved. B) A structure-based phylogenetic tree including Hpβ-clamp and its homologs used in this study. The structural alignment was carried out using STAMP and using percent identity to construct this phylogenetic tree. As seen in the tree, the Hpβ-clamp structure seems to have diverged from its homologs.

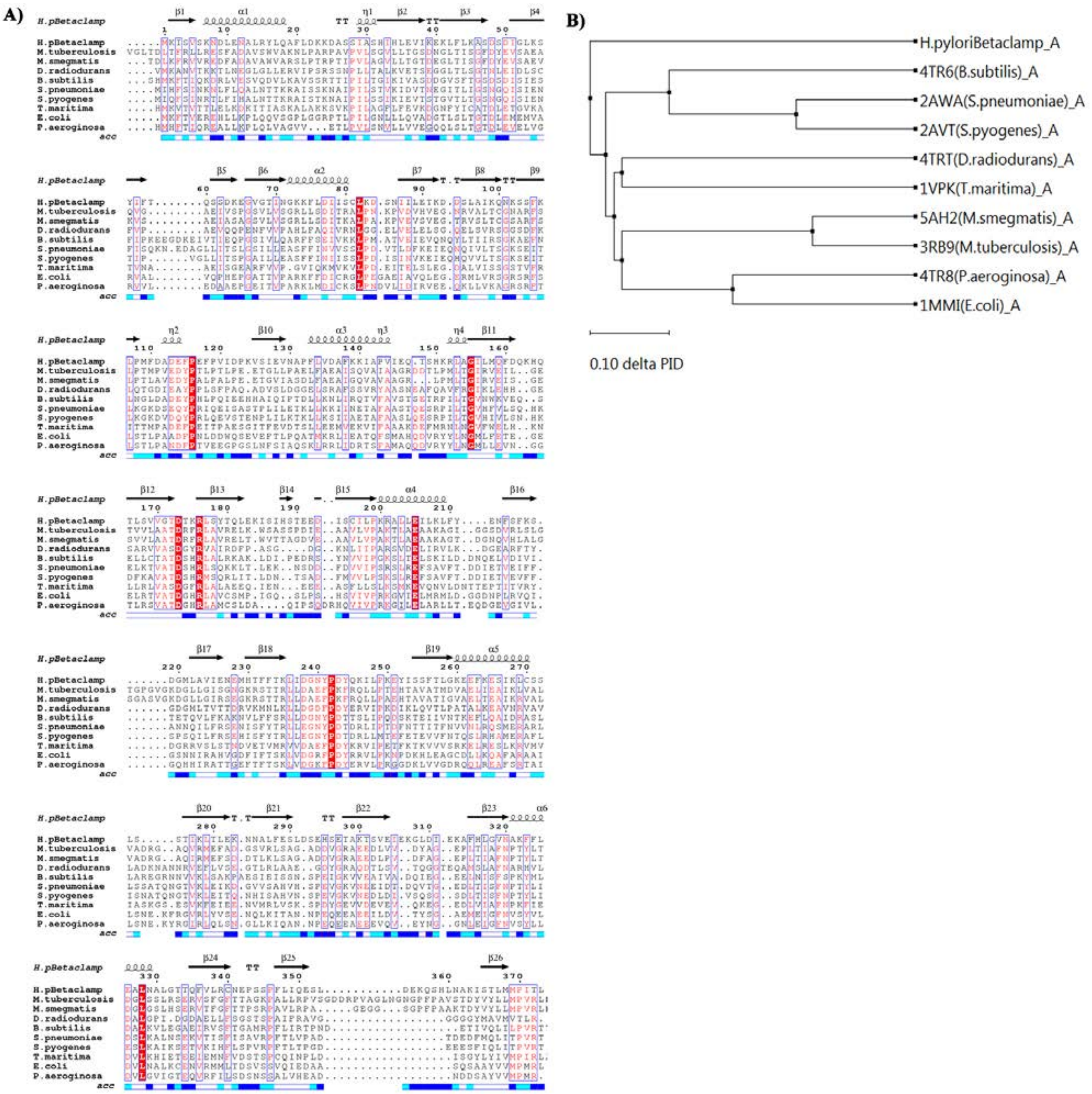
A)
B)
H.pyloriBetaclamp_A
4TR6(B.subtilis)_A
2AWA(S.pneumoniae)_A
2AVT(S.pyogenes)_A
4TRT(D.radiodurans)_A
1VPK(T.maritima)_A
5AH2(M.smegmatis)_A
3RB9(M.tuberculosis)_A
4TR8(P.aeroginosa)_A
1MMI(E.coli)_A
0.10 delta PID

Figure S.2 Cloning and expression profile of Hpβ-clamp. A) Plasmid PCR of Hpβ-clamp. M denotes the standard DNA marker, C is the negative control, and P1, P2, P3 and P4 are the plasmids from different colonies. The gene is 1122 bp long. B) GFC profile of Hpβ-clamp after being purified using Ni affinity column. The concentrated protein was loaded and eluted on Superdex G-200. The protein eluted at 72 ml as a dimer and 80 ml as monomer. C) SDS gel of the Hpβ-clamp protein; this gel indicated its size to be 42 KDa. Lanes L1 and L2 belong to different regions of the main peak (at 72 ml) and lane L3 belongs to a second peak (at 80 ml).

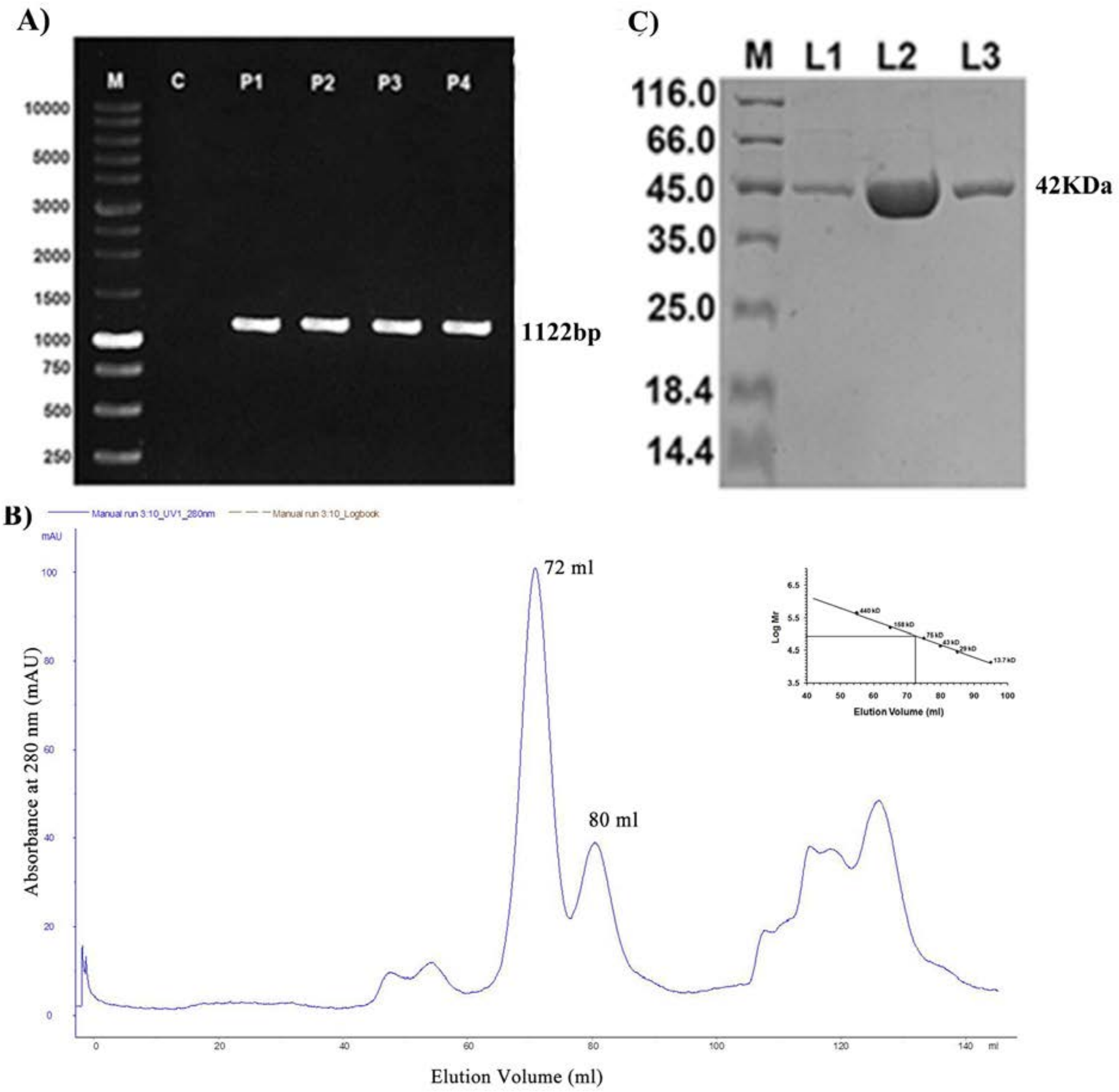

Figure S.3 Structural alignment of Hpβ-clamp domains. A) Structure-based sequence alignment of all three domains of Hpβ-clamp. In the sequence block, the residues colored white on a red background are completely conserved in the sequences shown, and the residues colored red on a white background are partially conserved. The bar below shows the accessible areas: blue, cyan, and white denote highly accessible, intermediately accessible and buried residues, respectively. B) A superposition of all three domains of Hpβ-clamp suggests all three domains to be structurally similar, except for some loops.

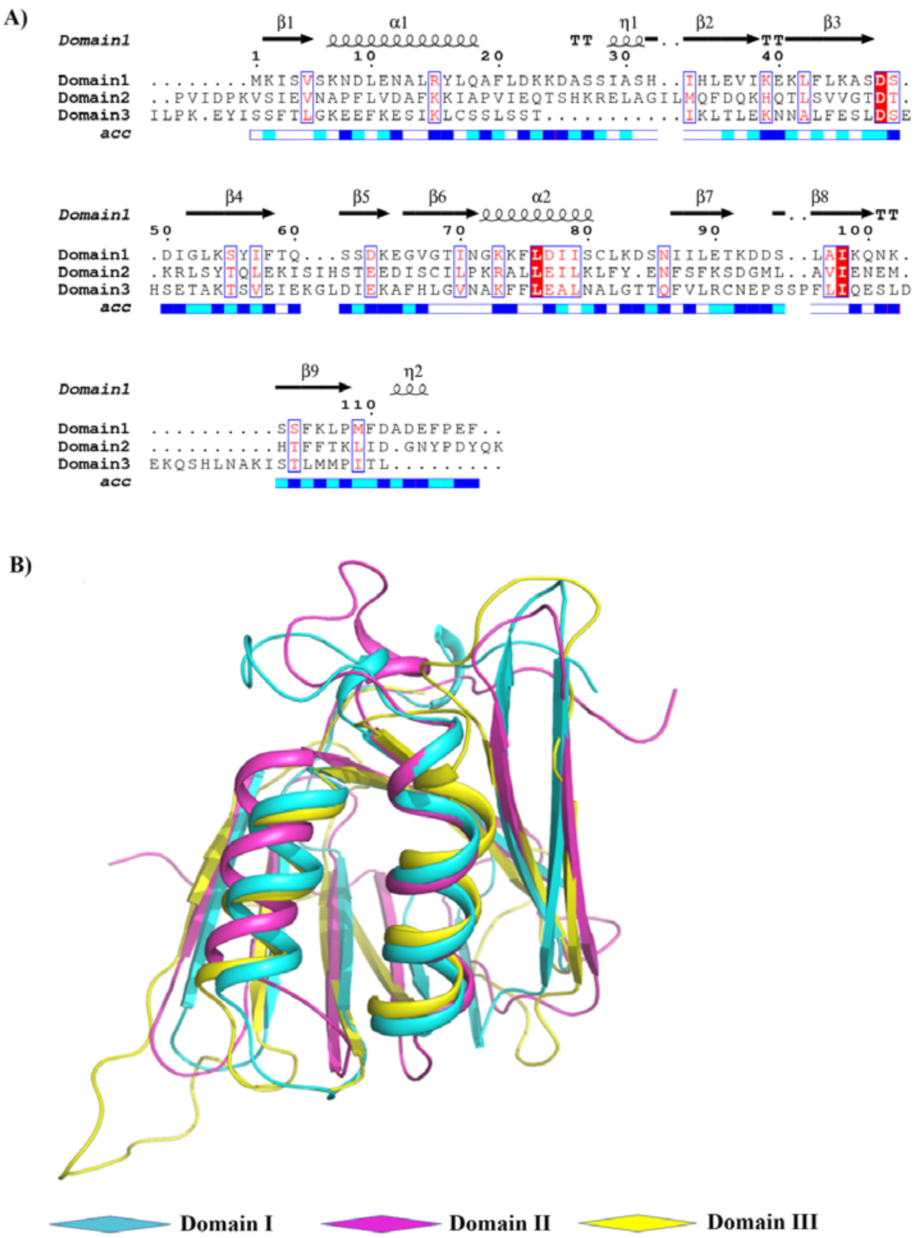

Figure S.4 Electrostatic surface potential of Hpβ-clamp and its homologs. The electrostatic surface potential was generated using the APBS plugin. The monomers of Hpβ-clamp and its homologs are shown with the DNA-binding surface of the monomer and dimeric interface. The DNA-binding surface of Hpβ-clamp was found to be similar to that of *E.coli* in that they both have a relatively large number of electropositive residues on the inner side of the ring, while β-clamps of other organisms have fewer electropositive residues, indicating only a moderate conservation of positively charged residues. The dimer interface of β-clamp of *H. pylori* was observed to be similar to those of *E.coli, M.tuberculosis* and *P.aeruginosa*, in that they consist of C-terminal electronegative residues and N-terminal electropositive residues, while β-clamp from *T.maritima* and *S.pyogenes* have an even distribution of both positive and negative charges.At the dimeric interface, the N- and C-termini were found to face each other to help link the monomers. This arrangement suggests that surface charges drive the interaction at the dimer interface.

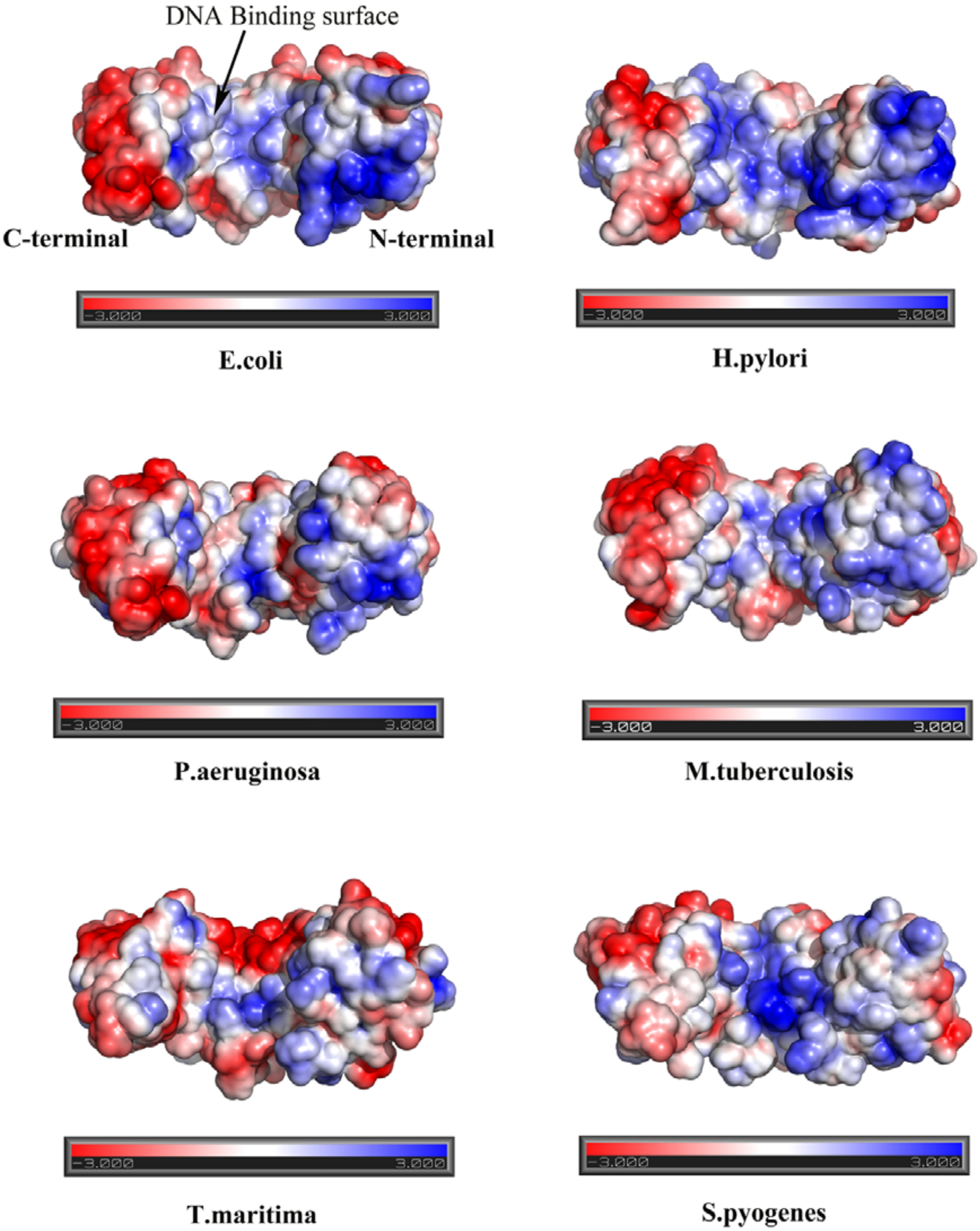
DNA Binding surface
C-terminal
N-terminal
-3.000
3.000
E.coli
H.pylori
P.aeruginosa
M.tuberculosis
T.maritima
S.pyogenes

Figure S.5 SAXS derived structural parameters for Hpβ-clamp, HpDNA ligase and Hpβ-clamp-ligase complex in solution. Plot of I(q) Vs q for β-clamp (A), DNA ligase (C) and mixture of β-clamp-ligase (in 1:1.25 ratio) (E) are shown in the figure. Pairwise interatomic distance distribution function P(R) plotted for β-clamp (B), Ligase (D) and mixture of β-clamp-ligase (F). The radius of gyration (Rg) calculated for β-clamp was 3.87 and Dmax 9.8nM. The shape resembles to flat disc. For ligase Rg calculated to be 4.73 and Dmax 14.5nM. This nearly resembles to long rod. However for the mixture of these two proteins, the Rg was calculated to be 6.3 with a Dmax 22nM which is quiet bigger compared to the Rg's calculated for the proteins alone suggesting the formation of complex.

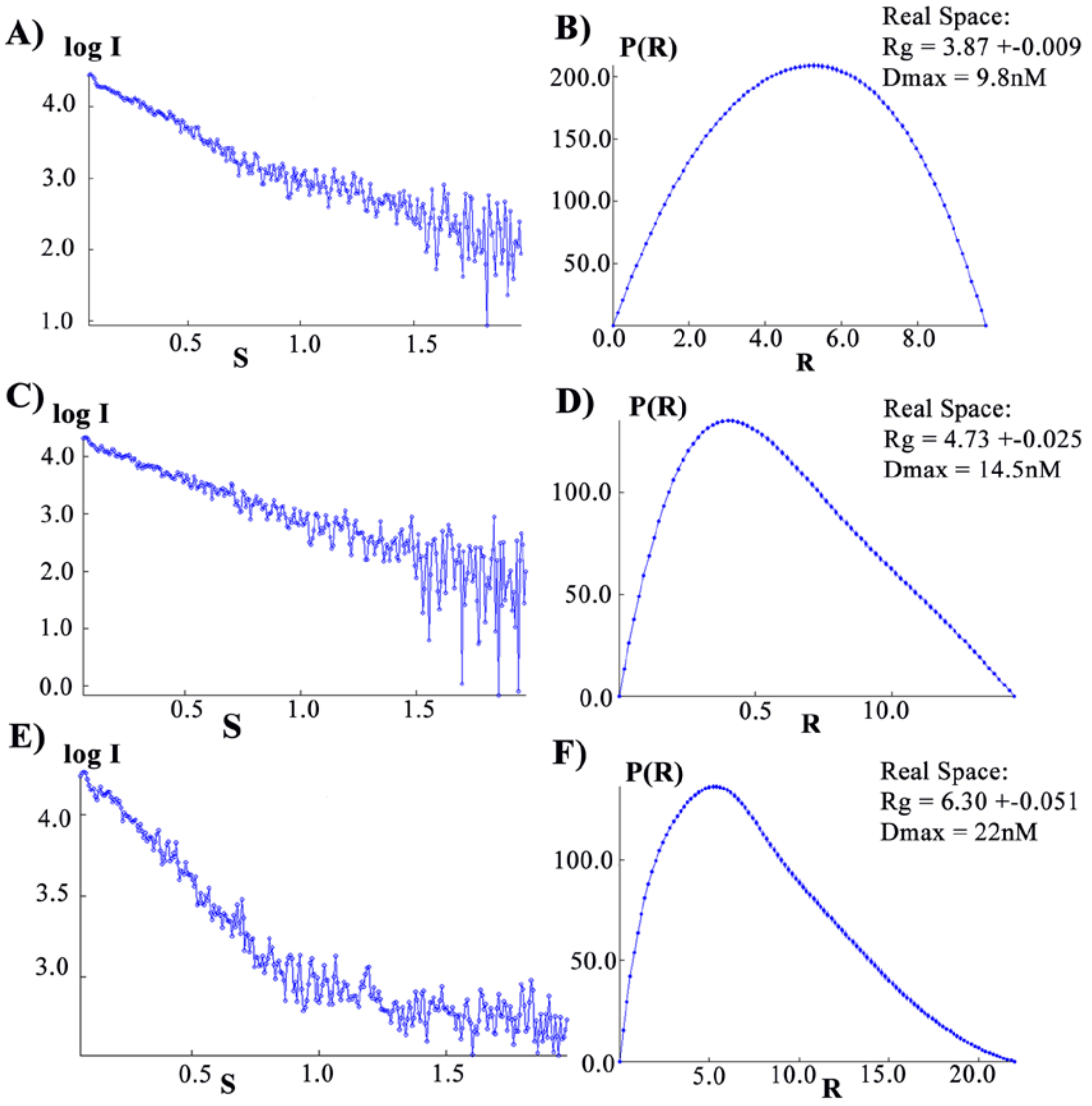

Figure S.6 Figure representing the changes in the residues after peptide binding. A) Surface representation of native beta clamp (no peptide bound). The area circled and arrow marked shows the loop region and residues that become displaced upon peptide binding so as to form a proper cleft. B) Surface representation of peptide-bound chain A of β-clamp. Upon peptide binding, the residues indicated by arrows were observed to become displaced a little bit to form a cleft receptive to the binding of a peptide. Residues Ile557, Leu560, and Phe561 from the peptide were observed to fit in the cleft. C) Surface representation of chain D bound to the peptide bound in similar fashion D) Structural alignment of native β-clamp (no bound peptide) (green) with chain A (pink) and chain D (red) of peptide-bound β-clamp. The figure shows major displacement of residues K176 and M370 with minor shifting in residues T173, T175, L178, I248, P347, P371, I372, T373, which allows the peptide to bind into the protein-binding cleft of beta clamp.

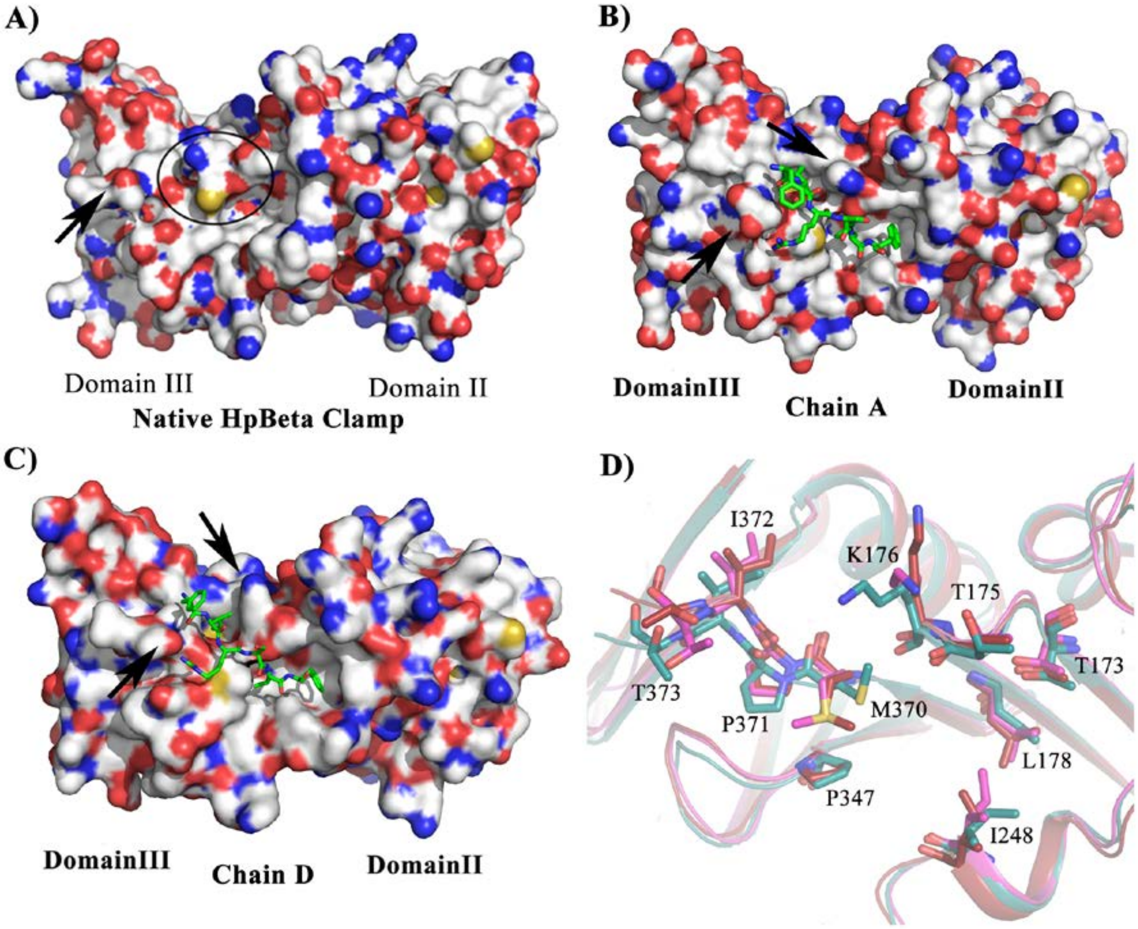

Figure S.7 Structural alignment of Hpβ-clamp with peptide-bound Ecβ-clamp and other homologs. A) Superimposition of Hpβ-clamp on peptide-bound Ecβ-clamp. The peptides superimposed here are from different co-crystal structures of Ecβ-clamp with its interacting partners. The peptide colored red is fromthe alpha subunit of DNA Pol III, wheat is from the delta subunit of DNA Pol III, violet from Pol II, and cyan fromPol IV. The superimposition was done to analyze the peptide-binding cleftbetween domain II and domain III. The groove of Hpβ-clamp appears to be very similar to that of Ecβ-clamp, with just small differences. B) In domain III, the loop with residues 295-301 in Hpβ-clampwas observed to be larger and extended more towards the peptide-binding sitethan was the loop in Ecβ-clamp. This loop may participate in peptide binding. C) In their respective crystal structures, Phe278 in domain III of Ecβ-clamp interacts with Glu3 of the peptide from the alpha subunit, and R279 of Ecβ-clamp interacts with Q69 of the delta subunit, whereas in Hpβ-clamp the corresponding residue is missing as the loop is shorter.

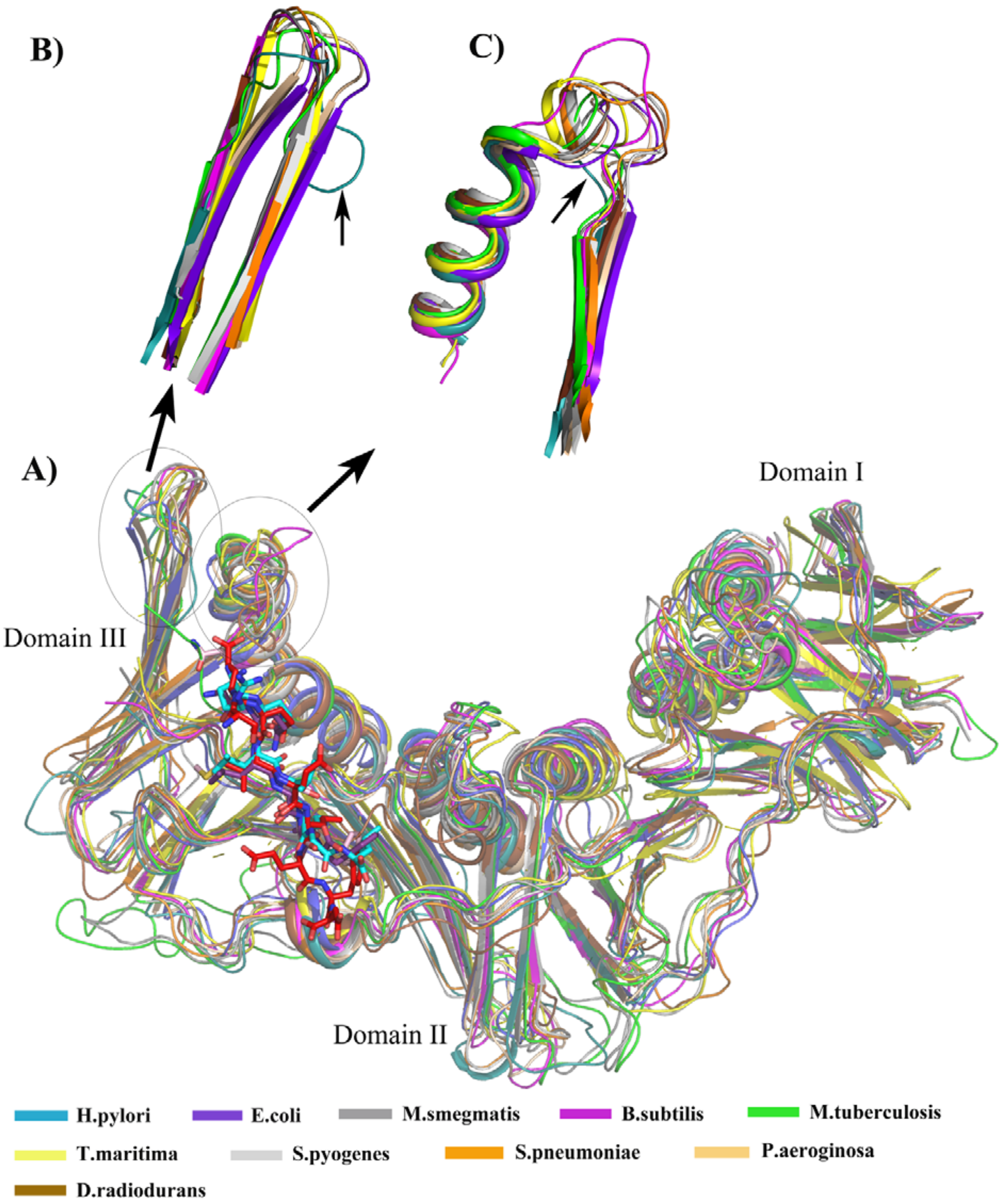
B)
C)
A)
Domain I
Domain III
Domain II
H.pylori
E.coli
M.smegmatis
B.subtilis
M.tuberculosis
T.maritima
S.pyogenes
S.pneumoniae
P.aeroginosa
D.radiodurans

Figure S.8 Cloning and expression profile of HpDNA ligase. A) Colony PCR of HpDNA ligase. The symbols M stands for the standard DNA marker,L1 to L9 are colony PCR results where L8 shows the positive colony. The gene is 1971bp long. B) SDS gel of HpDNA ligase after being purified using Ni-NTA; this gel indicated the size of the HpDNA ligase protein to be 73.9 KDa. The protein eluted at 30mM imidazole (elution 1) followed by 50mM (elution 2) and 80mM imidazole (elution 3). C) GFC profile of HpDNA ligase after being purified using Ni-NTA. Superdex G-200 was used for the purification. The protein eluted at 60 ml as a dimer.

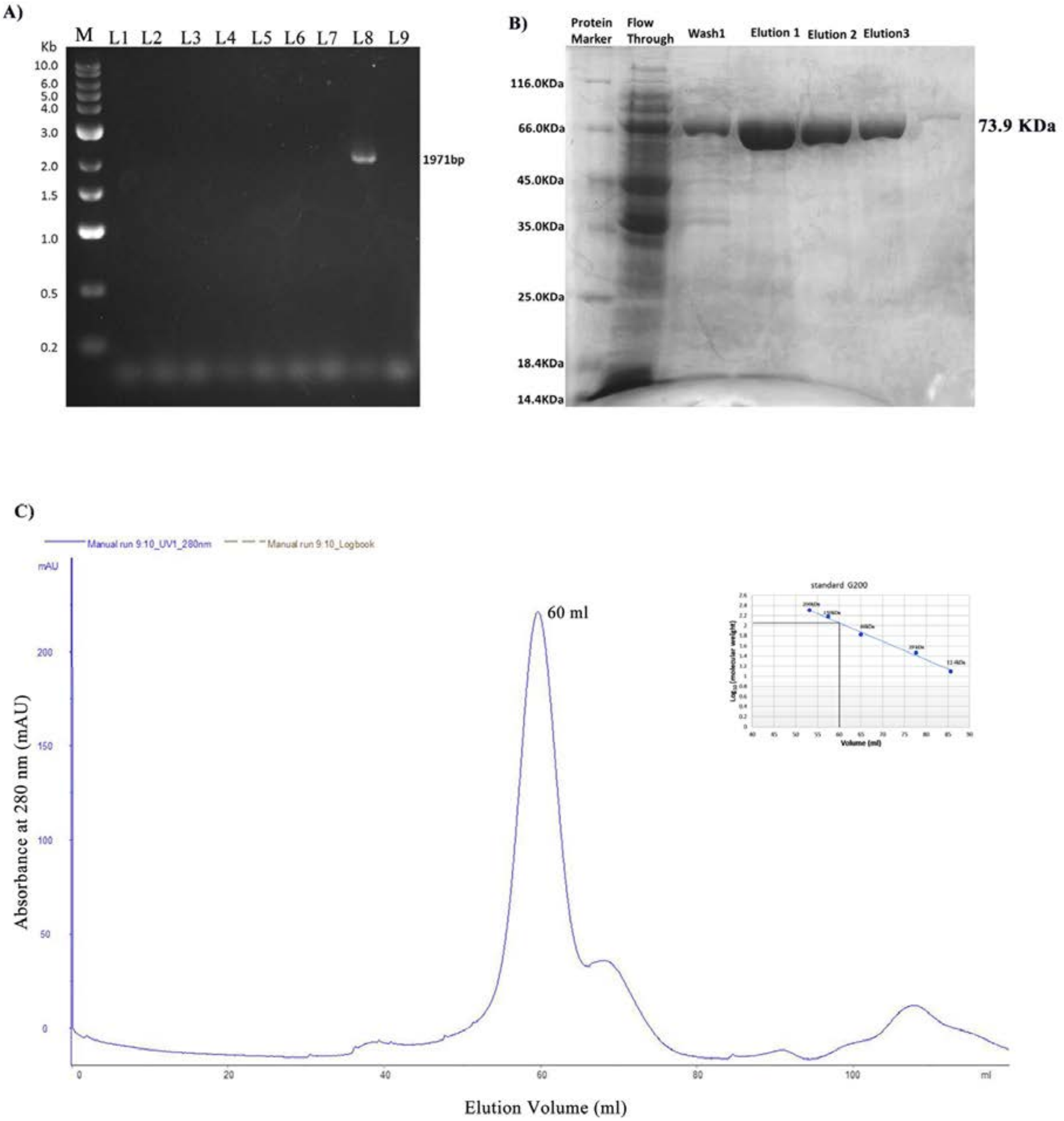

TABLE S.1

S.No.	Residues in Hpβ-clamp ChainA	Residues inHpβ-clamp ChainB
1.	Lys74	Leu274, Glu298, Glu295
2.	Asp77	Ser273
3.	Ile78	Ser273, Leu274, Leu270,
4.	Cys81	Leu270, Lys269, Ser273, Glu266
5.	Leu82	Leu270
6.	Lys83	Glu266
7.	Ser95	Ser297
8.	Lys102	Ser303, Thr302, Glu305, Val304, Lys301
9.	Ser103	Thr302, Lys301, Leu270, Lys301
10.	Ser104	Thr302, Lys301, Ala300, Thr299
11.	Phe105	Thr299, Ala300, Leu274, Leu270, Lys301
12.	Lys106	Thr299, Ser297, Glu298, Leu274
13.	Leu107	Ser297, Glu298, Leu274,
14.	Pro108	Glu298, Ser297

Table S.1 **Residues involved in the association between chain A and B to form the Hpβ-clamp dimer.**

TABLE S.2

Region	B-factor in *H.pylori*	B-factor in *E.coli*	B-factor in M.tuberculosis	B-factor in P.aerugin-osa	B-factor in B. Subtilis	B-factor in S.pneumo-niae	B-factoor in S. pyogenes	B-factor in T.maritima	B-factor in M.smegmatis	B-factor in D.radi-odurans
Mean B value	35.62	26.72	108.44	34.57	32.03	60.17	41.25	46.91	43.23	39.44
Domain I	32.07 (Residue 20-28)	73.90 (Residue 20-28)	127.60 (Residue 32-40)	46.7 (Residue 20-28)	41.9 (Residue 20-28)	71.07 (Residue 23-33)	27.37 (Residue 22-28)	64.1 (Residue 21-27)	46.9 (Residue 34-42)	48.1 (Residue 21-29)
Domain II	34.42 (Residue 210-214)	38.39 (Residue 206-213)	150.19 (Residue 217-223)	42.2 (Residue 207-213)	38.5 (Residue 215-221)	67.00 (Residue 215-221)	42.8 (Residue 215-221)	52.8 (Residue 207-212)	31.31 (Residue 219-227)	52.9 (Residue201-206)
Domain III	33.24 (Residue 274-277)	24.91 (Residue 273-279)	117.09 (Residue 290-296)	38.59 (Residue 274-280)	36.11 (Residue 280-290)	75.3 (Residue 283-290)	62.65 (Residue 283-290)	54.3 (Residue 274-280)	36.8 (Residue 293-298)	67.2 (Residue267-274)
	40.98 (Residue 292-300)	26.82 (Residue 295-301)	125.7 (residue 311-317)	39.4 (Residue 296-302)	29.2 (residue 306-310)	65.03 (Residue 305-309)	62.7 (Residue 305-309)	52.1 (Residue 295-299)	62.4 (Residue 310-313)	65.1 (Residue 291-294)
	45.15(Residue 355-365)	28.73 (Residue 351-354)	177.7 (Residue 368-391)	25.0 (Residue 351-358)	29.46 (Residue 362-368)	68.7 (Residue 361-367)	50.9 (Residue 361-367)	49.0 (Residue 350-355)	47.3 (Residue 365-385)	44.0 (Residue 346-353)

Table S.2 B-factor calculation for selected loop regions. The B-factor was calculated for the regions involved in DNA binding and peptide binding taking reference from E.coli against overall mean B-factor value for Hpβclamp and its homologs. Certain regions of Hpβ-clamp (specifically the loop regions in Domain I, II and III) shows variation in B-factor compared to other organisms.

TABLE S.3

**S.No.**	***E.coli* DNA interacting Residues**	**Corresponding residues to Ecoli DNA binding residues in**								
		H.pylori	***Mtb***	***S.pyogenes***	***S.pneumoniae***	***T.maritima***	***P.aeruginosa***	***B.subtilis***	***M.smegmatis***	***D.radiodur-ans***
**Residues from chain A**										
**1**	Glu8	Asn8	Glu20	Thr9	Asn9	Leu8	Glu8	Asp8	Glu18	Lys9
**2**	Leu11	Glu11	Ala23	Ile12	Leu12	Lys11	Leu11	Val11	Ala21	Asn12
**3**	Lys12	Asn12	Asp24	His13	Gln13	Asp12	Lys12	Glu12	Asp22	Glu13
**4**	Gln15	Arg15	Ser27	Asn16	Asn16	Thr15	Gln15	Gln15	Ala25	Gly16
**5**	Phe76	Leu76	Ser87	Ile80	Ile83	Gln76	Met76	Ser83	Ser85	Ala77
**6**	Arg80	Ser80	Arg91	Ser87	Ser87	Lys80	Lys80	Lys87	Lys89	Arg81
**7**	Asp208	Tyr212	Ile216	Thr216	Thr216	Asp206	Thr209	Asp216	Thr217	Lys204
**Residues from chain B**										
**1**	Arg24	Lys24	Arg36	Lys25	Lys25	Lys24	Arg24	Arg24	Arg34	Arg20
**2**	Arg73	Lys73	Arg84	Ser80	Ser80	Asp73	Arg73	Arg80	Arg82	His73
**3**	His148	Gln147	Arg157	Leu155	Thr155	Lys147	Gln148	Thr155	Arg155	Asn146
**4**	Gln149	Thr148	Asp158	Gln156	Gln156	Asp148	Gln149	Ser56	Asp156	Glu147
**5**	Arg197	Lys201	Ala205	Ser205	Ser205	Leu195	Arg198	Gly205	Ala206	Ala193
**6**	Lys198	Arg202	Lys206	Lys206	Arg206	Lys196	Lys199	Lys206	Lys207	Arg194

Table S.3 DNA binding residues of E.coli (determined using contact program in CCP4 with cut-off 4.5Å) and the equivalent residues in HpBclamp and its homologs.

TABLE S.4

S.No.	β-Clamp-Interacting Partners	Docking Score in *H.pylori*	Binding Energy (Kcal/mol)in*H.pylori*
1	C-terminal site of the DNA polymerase III alpha subunit (QGGNSLF in *H.pylori*))	-9.48	-8.35
2	NAD-dependent DNA ligase LigA (QEFIRSLF in *H.pylori*)	-8.896	-7.38

Table S.4 **Docking scores and binding energy values ofHpβ-clamp with its peptides from the Pol III alpha subunit and DNA ligase**. For Ecβ-clamp, no interaction site for NAD-dependent DNA ligase was detected.

TABLE S.5

Organism	PDB ID	Sequence similarity	Sequence Identity	RMSD r
S.pneumoniae	2AWA	44%	23%	2.46
T.maritima	1VPK	45%	22%	2.81
E. coli	2POL	44%	23%	1.70
S.pyogenes	2AVT	43%	21%	2.40
S.pneumoniae	2AWA	45%	23%	2.43
M.tuberculosis	3RB9	43%	20%	1.90
M.smegmatis	5AH2	45%	23%	1.89
P.aeroginosa	4TR8	46%	21%	1.55
D.radiodurans	4TRT	45%	22%	2.55

Table S.5 **Sequence similarity and structural deviation of Hpβ-clamp from its homologs**. The table suggests that Hpβ-clamp is more closer to *P.aeroginosa* as compared to other organisms
